# Clinical Instruction in Chicago

**Published:** 1866-12

**Authors:** 


### CLINICAL INSTRUCTION IN CHICAGO.

The increased advantages for practical instruction at the bedside, which this city now affords, cannot fail to mark Chicago as an important point in our country for the character of its medical institutions.

The Mercy and Marine Hospitals afforded, for many years, almost the only facilities in the city for clinical observation. To these institutions many hundred of practitioners in the land will look with deep satisfaction, in remembrance of the knowledge of disease they gained under the guidance of instructors whose names are familiar in every village of the Northwest.

These hospitals still afford material of the greatest value for the student. But the opening of the County Hospital at the close of the past year, was the commencement of an important era in the history of medical education in Chicago. This hospital now contains nearly one hundred patients, presenting examples of almost every form of disease. Without doubt the number of patients will increase each year in proportion to the rapid growth of the city.

The recent very great improvements in the accommodations for the medical class, have added much, not only to the comfort of students, but to their means of observing operations, and of inspecting the different cases of diseases, and the *post mortem* specimens.

The number of autopsies is quite large, and presents a very important feature in the courses of instruction at the hospital.

The following synopsis of the annual report of the Medical Board will interest our readers:

Since the organization of the hospital, in January last, the number of patients under treatment has been 807. Of these, the number admitted into the medical wards was 505, into the surgical, 258; into the Eye and Ear Department, 44. From the medical wards the number discharged, cured, was 326; number relieved, 50; number of deaths, 87; remaining, 42. From the surgical wards the number discharged after cure was 208; relieved, 10; deaths, 10; remaining, 30; total, 257.

From the Eye and Ear Department there have been discharged, after cure, 27; relieved, 5; transferred to medical department, 1; absconded, 3; remaining, 8; total, 44. Number of births in the lying-in rooms 34.

During the month of October a dispensary for the relief of the outside poor was opened in the basement of the hospital, and the number thus supplied was 85. The dispensary is open every day during the week, Sundays excepted, from 1 to 2 o'clock in the afternoon, for medical and surgical patients. Patients with disease of the eye and ear are admitted at 11 o'clock in the morning.

The high rate of mortality among the inmates is accounted for by a combination of causes, which in public institutions like this, from which the homeless victims of incurable diseases cannot be excluded, always operate to swell the lists of deaths beyond the figures which obtain in those hospitals where only acute diseases are treated. Of the deaths which occurred in the medical wards, 45 were of chronic incurable diseases. Eight inmates have died of cholera, and about twenty persons have been admitted in a moribund state whose death occurred in a few hours after being received.

The hospital is well provided with ample means for service, and is throughout in most excellent condition.

The college clinics also afford facilities for instruction by no means insignificant, and are fully attended by the different classes in the city.

In addition to these institutions may be mentioned the Chicago Charitable Eye and Ear Infirmary, which is open for all students, who wish to gain a knowledge of the diagnosis and treatment of ophthalmic and aural diseases.

The facilities for instruction thus offered in these departments of medicine and surgery, are probably inferior to those of no city in the Northwest. More than five hundred patients, with diseases of the eye and ear, applied to the Infirmary for treatment during the past year.

